# Loss of *TG‐Interacting Factor 1* decreases survival in mouse models of myeloid leukaemia

**DOI:** 10.1111/jcmm.15977

**Published:** 2020-10-15

**Authors:** Ling Yan, Utpal P. Davé, Michael Engel, Stephen J. Brandt, Rizwan Hamid

**Affiliations:** ^1^ Departments of Pediatrics Vanderbilt University Medical Center Nashville TN USA; ^2^ Department of Medicine, and Microbiology and Immunology Indiana University Indianapolis IN USA; ^3^ Department of Pediatrics University of Virginia Charlottesville VA USA; ^4^ Departments of Medicine Vanderbilt University Medical Center Nashville TN USA

**Keywords:** acute myeloid leukaemia, bone marrow cells, chronic myeloid leukaemia, leukaemia‐initiating cells, TG‐Interacting Factor 1

## Abstract

*TG‐Interacting Factor 1* (*Tgif1*) affects proliferation and differentiation of myeloid cells and regulates self‐renewal of haematopoietic stem cells (HSCs). To determine its impact on leukaemic haematopoiesis, we induced acute or chronic myeloid leukaemias (AML or CML) in mice by enforced expression of *MLL‐AF9* or *BCR‐ABL*, respectively, in *Tgif1^+/+^* or *Tgif1^−/−^* haematopoietic stem and progenitor cells (HSPCs) and transplanted them into syngeneic recipients. We find that loss of *Tgif1* accelerates leukaemic progression and shortens survival in mice with either AML or CML. Leukaemia‐initiating cells (LICs) occur with higher frequency in AML among mice transplanted with *MLL‐AF9*‐transduced *Tgif1^−/−^* HSPCs than with *Tgif1^+/+^* BMCs. Moreover, AML in mice generated with *Tgif1^−/−^* HSPCs are chemotherapy resistant and relapse more rapidly than those whose AML arose in *Tgif1^+/+^* HSPCs. Whole transcriptome analysis shows significant alterations in gene expression profiles associated with transforming growth factor‐beta (TGF‐beta) and retinoic acid (RA) signalling pathways because of *Tgif1* loss. These findings indicate that *Tgif1* has a protective role in myeloid leukaemia initiation and progression, and its anti‐leukaemic contributions are connected to TGF‐beta‐ and RA‐driven functions.

## 1 INTRODUCTION

*TG‐Interacting Factor1* (*TGIF1*) belongs to the three‐amino acid loop extension (TALE) family of homeodomain proteins^1^, ^2^, ^3^, ^4^ and functions as a corepressor of retinoic acid (RA) and transforming growth factor‐β (TGF‐β)‐stimulated transcription. It does so by interfering with retinoid X receptor (RXR) binding to DNA and by recruiting general corepressors,^5^ histone deacetylases (HDACs) to the TGF‐β signalling intermediate Smad2,^2^, ^3^, ^4^, ^6^ respectively. TGIF1 can also inhibit transcription directly through binding to a TG‐rich sequence element *via* its homeobox domain. Inactivating mutations in *TGIF1* cause autosomal dominant holoprosencephaly,^7^, ^8^, ^9^, ^10^ the most common inherited defect in forebrain development in humans.

In addition to its role in forebrain development, *TGIF1* affects proliferation and differentiation of myeloid lineage cells by regulating cell cycle progression.^11^ Also, we have shown that *Tgif1* regulates self‐renewal of haematopoietic stem cells (HSCs). Loss of *Tgif1* in mice increases quiescence in bone marrow HSCs and enhances long‐term repopulating activity without an effect on steady‐state haematopoiesis.^12^ In MLL‐rearranged AML, which is typically aggressive and portends a poor prognosis, *TGIF1* expression is decreased compared to AML cases characterized by other molecular lesions.^13^ Moreover, enforced expression of *TGIF1* in MLL‐AF9‐transduced leukaemia cells influences transcriptional networks regulated by MEIS1, another TALE family member, while the *TGIF1*:*MEIS1* ratio predicts AML survival. These data suggest that altered TGIF1 function could alter outcomes for AML patients.

Here, we present data showing that in addition to its role in normal HSCs, *Tgif1* also affects leukaemia‐initiating cell (LIC) function in AML. Loss of *Tgif1* increases LIC frequency in both acute and chronic myeloid leukaemia mouse models, resulting in earlier disease relapse, reduced survival and treatment resistance.

## 2 MATERIALS AND METHODS

### 2.1 Mice and plasmids

Mice with a *Tgif1* null mutation have been previously described.^12^ C57BL/6J mice were purchased from the Jackson Laboratories (Bar Harbor, ME). All animal experiments were approved by the Animal Care and Use Committees of Vanderbilt University and University of Virginia. MSCV‐MLL‐AF9‐IRES‐GFP vector was provided by Dr Scott Armstrong (Boston Children's Hospital, Boston, MA).

### 2.2 Retrovirus production

Retroviral vectors were transfected into Phoenix ecotropic retroviral packaging cells using Lipofectamine 2000 according to the manufacturer's instructions. Forty‐eight hours after transfection, viral supernatants were collected, filtered and stored at −80°C.

### 2.3 Induction of myeloid leukaemia in mice

The MSCV‐MLL‐AF9‐IRES‐GFP retrovirus was used to induce AML in mice. Bone marrow cells were flushed from the tibias and femurs of *Tgif1^+/+^* or *Tgif1^−/−^* mice and lineage marker‐negative (Lin^‐^) cells were enriched using the Mouse Lineage Cell Depletion kit (Miltenyi Biotec). Lin^‐^ cells purified from *Tgif1^+/+^* or *Tgif1^−/−^* mice were then transduced using low‐speed centrifugation (spinoculation), with MSCV‐MLL‐AF9‐IRES‐GFP retrovirus in the presence of 8 μg/mL polybrene at 1350g × 45 minutes (32°C). Transduced *Tgif1^+/+^* or *Tgif1^−/−^* Lin^‐^ cells were resuspended in phosphate‐buffered saline (PBS), and 1 × 10^5^ cells were injected intravenously into sub‐lethally irradiated (4.5 Gy) *Tgif1^+/+^* or *Tgif1^−/−^* recipient mice, respectively. Reconstitution of transduced cells in recipient mice was evaluated by monitoring GFP expression by flow cytometry analysis at 1‐2 weeks intervals following transplantation. Recipient mice were killed for analysis when they developed palpable splenomegaly or appeared ill. Bone marrow and spleen cells from leukaemic mice were isolated and stored frozen for later use.

MSCV‐BCR/ABL‐IRES‐GFP retrovirus was used to induce CML in mice. Bone marrow cells were flushed from tibias and femurs of *Tgif1^+/+^*, *Tgif1^−/−^* or *Tgif1^±^* mice, and single‐cell suspensions of Lin^‐^ c‐Kit^+^ cells were obtained using the Mouse Lineage Cell Depletion kit and CD117 microbeads (Miltenyi Biotec, San Diego, CA). Lin^‐^ c‐Kit^+^ cells were spinoculated with MSCV‐BCR/ABL‐IRES‐GFP retrovirus in the presence of 8 μg/mL polybrene for 45 minutes at 1350 g (32°C). Transduced cells were resuspended in PBS, and 8 × 10^4^ cells were injected intravenously into lethally or sub‐lethally irradiated C57BL/6J recipient mice. Reconstitution of transduced cells in recipient mice was evaluated by flow cytometry analysis of GFP expression two weeks after transplantation. Mice were monitored for disease development, at which time they were killed.

### 2.4 Quantification of leukaemia‐initiating cell frequency

Leukaemia cells from spleens of three *Tgif1^+/+^* or three *Tgif1^−/−^* mice with AML induced with MSCV‐MLL‐AF9‐IRES‐GFP retrovirus were pooled, and 1 × 10^6^, 1 × 10^5^, 1 × 10^4^, 1 × 10^3^, 100 and 50 cells were transplanted into sub‐lethally irradiated recipients. Animals were killed when they became visibly ill, and the development of leukaemia was confirmed. LIC frequency was determined by limiting dilution analysis with the ELDA (for Extreme Limiting Dilution Analysis) software package.^14^

### 2.5 Chemotherapy studies in AML mice

For treatment studies, 2 × 10^3^ splenic leukaemia cells from *Tgif1^+/+^* or *Tgif1^−/−^* mice were transplanted into sub‐lethally irradiated recipients by tail vein injection. Two weeks after transplant, mice were treated by intraperitoneal injection with 100 mg/kg cytarabine once per day for five days and 3 mg/kg doxorubicin for three days. Mice were monitored closely for disease development, and GFP expression was evaluated weekly by flow cytometry analysis. Animals were killed when they appeared ill.

### 2.6 RNA sequencing (RNA‐seq) analysis of AML cells

RNA was extracted from sorted populations of GFP‐expressing leukaemia cells isolated from the bone marrow or spleen of *Tgif1^+/+^* or *Tgif1^−/−^* mice with AML. RNA‐seq analysis was performed on leukaemia cells from three separate mice for each condition. Sequencing of RNA and subsequent bioinformatic analyses was performed by the Vanderbilt Technologies for Advanced Genomics (or VANTAGE) Shared Resource. Sequencing data were annotated with the University of California, Santa Cruz Genome Browser (https://genome.ucsc.edu/), and the TopHat and Cufflinks software packages were used for transcript alignment and quantification of gene expression, respectively. Gene set enrichment analysis^15^ was carried out using the default weighted enrichment statistic to test whether differentially expressed genes (between *Tgif1^+/+^* and *Tgif1^−/−^* LSK cells) were randomly distributed or enriched at the top or bottom of the gene list. A false discovery rate of ≤0.25 was considered significant. Gene ontology analysis was performed with Ingenuity Pathway Analysis (http://www.ingenuity.com/) to group functionally related genes and assign them to biological pathways.

## 3 RESULTS

### 3.1 Tgif1 gene loss decreased survival in an experimental model of acute myeloid leukaemia

**FIGURE 1:**
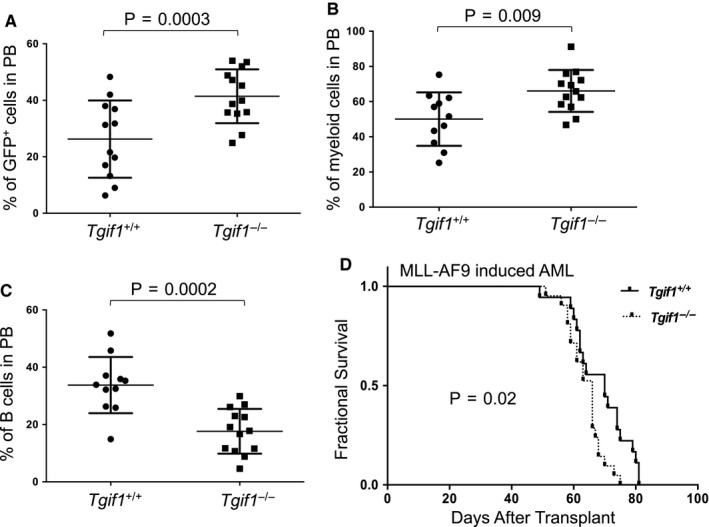
MLL‐AF9‐induced AML in *Tgif1^−/−^* mice was more aggressive with a shorter survival time than that in *Tgif1^+/+^* mice. Lin^‐^ bone marrow cells (Lin^‐^HSPCs) from *Tgif1^+/+^ or Tgif1^−/−^* mice were transduced with MLL‐AF9‐GFP retrovirus and transduced cells transplanted into sub‐lethally irradiated *Tgif1^+/+^* and *Tgif1^−/−^* recipient mice, respectively. Six weeks after transplant, the percentages of A, GFP^+^ cells; B, myeloid cells (CD11b^+^ and Gr‐1^+^) and C, B‐lymphocytes (B220^+^) in peripheral blood (PB) were analysed by flow cytometry. D, Kaplan‐Meier survival curves for indicated mice in MLL‐AF9‐induced AML

We have previously shown that *Tgif1* deletion promotes quiescence and self‐renewal activity in haematopoietic stem cells (HSC) in mice without disturbing steady‐state haematopoiesis.^12^ Studies have also shown *TGIF1* expression is decreased in MLL‐rearranged AML patients, favouring an anti‐leukaemic role.^13^ Here, we sought to determine directly whether *Tgif1* expression also affects leukaemic haematopoiesis in mice. We induced AML in mice by introduction of an *MLL‐AF9* fusion cDNA in lineage‐negative haematopoietic stem and progenitor cells (Lin^‐^HSPCs) from *Tgif1^+/+^* and *Tgif1^−/−^* mice. Transduced cells were transplanted into sub‐lethally irradiated recipients via tail vein injection. Development and progression of leukaemia in recipient mice was then monitored by flow cytometry analysis for green fluorescent protein (GFP)‐expressing nucleated cells in peripheral blood. Six weeks after transplant, mice that received *MLL‐AF9*‐transduced *Tgif1^−/−^* HSPCs showed higher numbers of GFP^+^ myeloid (CD11b^+^ and Gr‐1^+^) cells (Figure 1A,B) but lower numbers of B cells (Figure 1C) compared to those receiving similarly transduced *Tgif1^+/+^* HSPCs. Necropsy showed that all mice with circulating donor‐derived leukaemia cells had splenomegaly, with massive infiltration of myeloblasts observed in both spleen and bone marrow (data not shown). These data suggest that *Tgif1^−/−^* HSPCs are more prone to AML compared to those with *Tgif1^+/+^* HSPCs upon enforced expression of MLL‐AF9 fusion protein.

Kaplan‐Meier analysis showed that mice transplanted with MLL‐AF9‐transduced *Tgif1^−/−^* HSPCs survived for a shorter time than mice transplanted with transduced *Tgif1^+/+^* HSPCs (Figure 1D). As mice transplanted with either *Tgif1^+/+^* or *Tgif1^−/−^* HSPCs succumbed to leukaemia with very short latency, the aggressive nature of this leukaemia almost certainly underestimates the impact of *Tgif*1 loss on survival. Regardless, these results indicate loss of *Tgif1* accelerates progression of MLL‐AF9‐induced AML in mice.

#### 3.1.1 Tgif1 loss increases leukaemia‐initiating cell (LIC) frequency in MLL‐AF9‐induced AML

**FIGURE 2:**
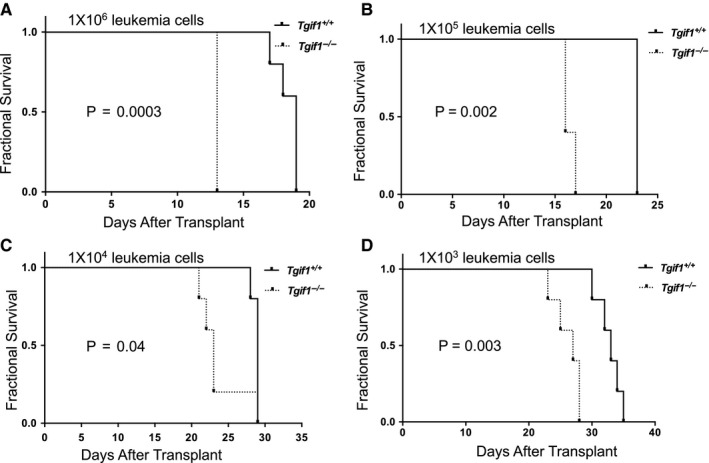
LSC frequency was increased in *Tgif1^−/−^* mice with AML compared to *Tgif1* ^+/+^ mice. Lin^‐^ bone marrow cells (Lin^‐^HSPCs) from *Tgif1^+/+^* and *Tgif1^−/−^* mice were transduced with MLL‐AF9‐GFP retrovirus and transduced cells transplanted into sub‐lethally irradiated *Tgif1^+/+^* and *Tgif1^−/−^* recipients, respectively. At a predetermined time, serial dilutions of spleen cells from mice with leukaemia then collected and transplanted into secondary recipients. Kaplan‐Meier analysis of survival is plotted as a function of number of transplanted spleen cells. A, 1 × 10^6^; B, 1 × 10^5^; C, 1 × 10^4^; D, 1 × 10^3^

LICs are crucial for maintenance of AML in vivo.^16^, ^17^, ^18^ Therefore, one possible explanation for shorter survival seen with *Tgif1* loss could be increased numbers of LICs. To explore this question, we carried out limiting dilution analysis to quantify LIC functionally by transplanting serial numbers of MLL‐AF9‐transduced *Tgif1^+/+^* or *Tgif1^−/−^* spleen cells from primary recipients into non‐conditioned wild‐type C57BL/6J mice. Indeed, our data showed a twofold higher frequency of LICs in *Tgif1^−/−^* mice with AML (1 in 125) (95% CI, lower: 1 in 279 and higher: 1 in 56.5) than in *Tgif1^+/+^* mice (1 in 250) (95% CI, lower: 1 in 603 and higher: 1 in 103.6). These data suggest that *Tgif1^−/−^* leukaemic populations were enriched in LIC number, function or both. Consistent with this notion, mice transplanted with *MLL‐AF9*‐transduced *Tgif1^−/−^* leukaemia cells over a range of doses had inferior survival compared to those with similarly transplanted *Tgif1^+/+^* cells (Figure 2A‐D).

#### 3.1.2 Tgif1 loss results in earlier relapse and more aggressive disease

**FIGURE 3:**
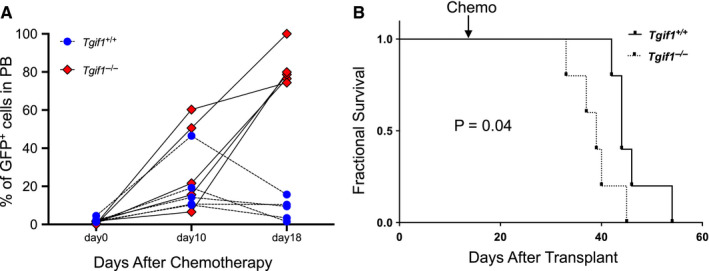
AML in *Tgif1^−/−^* mice progressed earlier after chemotherapy than in *Tgif1^+/+^* AML mice. Sub‐lethally irradiated C57BL/6 mice were transplanted with spleen cells from *Tgif1^+/+^* or *Tgif1^−/−^* mice with established MLL‐AF9‐induced AML, and two weeks following transplant, recipient mice were treated with doxorubicin for 3 d and cytarabine for 5 d by intraperitoneal injection. A, Percentage of GFP^+^ cells in peripheral blood were analysed using flow cytometry at day 0, day 10 and day18 after chemotherapy. B, Kaplan‐Meier analysis of survival of *Tgif1^+/+^* and *Tgif1^−/−^* mice with AML after chemotherapy

Next, we sought to investigate whether *Tgif1* expression is correlated with time to AML relapse and/or overall survival after treatment with conventional agents used in AML. To simulate the clinical setting, we transplanted *Tgif1^+/+^* and *Tgif1^−/−^*, *MLL‐AF9*‐transformed‐spleen cells from mice with established leukaemia into sub‐lethally irradiated recipients and treated these mice with cytarabine and doxorubicin chemotherapy as described.^19^ Our data showed that GFP‐expressing cells appeared earlier and with higher numbers in peripheral blood of mice transplanted with *Tgif1^−/−^* leukaemia cells than mice transplanted with *Tgif1^+/+^* leukaemia cells (Figure 3A). While chemotherapy treatment extended survival of both *Tgif1^+/+^* and *Tgif1^−/−^* mice with AML (compare Figure 3B to Figure 2D), mice transplanted with *Tgif1^−/−^* leukaemia cells still had shorter survival than mice transplanted with *Tgif1^+/+^* leukaemia cells (Figure 3B). Although selection at the level of a stem cell is not proven by these data, the differences in latency of disease and response to chemotherapy, we posit, are because of the higher frequency and/or greater fitness of LICs and more rapid expansion of leukaemia in *Tgif1^−/−^* mice.

#### 3.1.3 Tgif1 loss decreases survival in a model of chronic myeloid leukaemia

**FIGURE 4:**
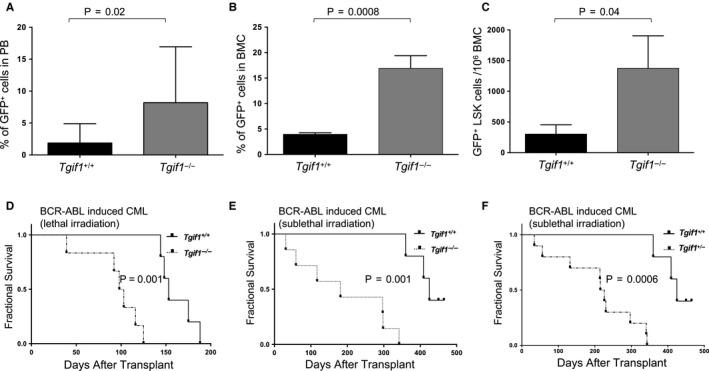
Mice with *Tgif1^−/−^* HSPCs developed more aggressive disease and had inferior survival compared to those with *Tgif1^+/+^* HSPCs in BCR‐ABL‐induced CML. Enriched Lin^‐^ c‐Kit^+^ cells from *Tgif1^+/+^, Tgif1^−/−^* or *Tgif1^±^* bone marrow cells (HSPCs) were transduced with BCR‐ABL‐GFP retrovirus and transduced cells transplanted into lethally or sub‐lethally irradiated C57BL/6J recipients to induce CML. Six weeks after transplant, flow cytometry was used to analyse A, percentage of GFP^+^ cells in PB; B, percentage of GFP^+^ cells in bone marrow; C, number of GFP^+^ LSK cells in bone marrow. Recipient mice were D, lethally irradiated; E and F, sub‐lethally irradiated in fractional survival curves

We next investigated whether *Tgif1* loss also affects leukaemic progression in a mouse model of CML. CML arguably has the best evidence supporting the existence of a malignant stem cell. To that end, Lin^‐^ HSPCs from *Tgif1^+/+^* and *Tgif1^−/−^* mice were transduced with the *BCR‐ABL‐GFP*‐expressing retrovirus and transplanted into sub‐lethally or lethally irradiated recipients. After transplant, the kinetics of leukaemic progression in recipient mice was monitored using GFP‐expressing myeloid cells in peripheral blood (PB). Six weeks after transplant, mice receiving *BCR‐ABL‐GFP*‐expressing *Tgif1^−/−^* cells showed higher numbers of GFP^+^ cells in PB and HSPCs, including the Lin^‐^Sca1^+^c‐Kit^+^ (LSK) population enriched with haematopoietic stem and progenitor cells, compared to *Tgif1^+/+^* mice (Figure 4A‐C, respectively). Kaplan‐Meier analysis revealed that mice transplanted with *BCR‐ABL‐GFP*‐transduced *Tgif1^−/−^* HSPCs showed significantly shorter survival than mice transplanted with *BCR‐ABL‐GFP*‐transduced *Tgif1* ^+/+^ HSPCs (Figure 4D). When recipient mice were conditioned with a lower dose of radiation, leukaemia developed with a longer latency; however, *Tgif1* genotype still significantly impacted survival (Figure 4E). Interestingly, mice receiving *BCR‐ABL*‐transduced heterozygous (*Tgif1^±^*) HSPCs also had shorter survival (Figure 4F) paralleling the greater long‐term repopulating ability of *Tgif1^±^* HSCs compared to *Tgif1^+/+^* HSCs.^12^ Collectively, these data are compatible with mouse AML data and suggest *Tgif1* expression impacts survival in CML.

#### 3.1.4 Tgif1 loss affects multiple transcriptional networks in AML

To gain insight into the biological and molecular pathways affected by *Tgif1* gene loss, we compared global gene expression profiles in *Tgif1^−/−^* and *Tgif1^+/+^* myeloid leukaemia cells by unbiased mRNA sequencing. We identified at least 45 genes that were differentially expressed in these two populations—33 of these were up‐regulated and 12 were down‐regulated in *Tgif1^−/−^* relative to *Tgif1^+/+^* leukaemia cells (Table 1).

**TABLE 1:** Differentially expressed genes in *Tgif1^−/−^* vs *Tgif1^+/+^* leukaemic cells

ID	Symbol	Location	Log ratio
Slc15a2	SLC15A2	Plasma Membrane	−7.651
Eps8l1	EPS8L1	Cytoplasm	−7.587
Dlgap1	DLGAP1	Plasma Membrane	−3.719
Gdf3	GDF3	Extracellular Space	−3.658
Camk2b	CAMK2B	Cytoplasm	−3.619
Col19a1	COL19A1	Extracellular Space	−3.488
Kcnq5	KCNQ5	Plasma Membrane	−2.727
Vill	VILL	Cytoplasm	−2.499
Cgnl1	CGNL1	Plasma Membrane	−1.938
Cdkn2c	CDKN2C	Nucleus	−1.649
Gm15448	LILRB3	Plasma Membrane	−1.362
Selm	SELM	Cytoplasm	−0.761
Ly75	LY75	Plasma Membrane	0.652
Anxa3	ANXA3	Cytoplasm	0.714
Agap1	AGAP1	Cytoplasm	0.742
Ttc21a	TTC21A	Extracellular Space	0.967
Pip5k1b	PIP5K1B	Cytoplasm	0.976
B4galt6	B4GALT6	Cytoplasm	1.029
Ptgs1	PTGS1	Cytoplasm	1.045
Olfm4	OLFM4	Extracellular Space	1.123
Dsp	DSP	Plasma Membrane	1.212
Gfi1	GFI1	Nucleus	1.278
Plekha6	Plekha6	Other	1.291
Sept5	SEPT5	Cytoplasm	1.301
Optn	OPTN	Cytoplasm	1.325
Kcnh7	KCNH7	Plasma Membrane	1.417
Fcnb	FCN1	Extracellular Space	1.427
S100a8	S100A8	Cytoplasm	1.503
Gca	GCA	Cytoplasm	1.575
Serpine2	SERPINE2	Extracellular Space	1.875
Camp	CAMP	Cytoplasm	1.940
Serpine1	SERPINE1	Extracellular Space	2.160
Retnlg	Retnlg	Extracellular Space	2.167
Cd74	CD74	Plasma Membrane	2.609
C1qb	C1QB	Extracellular Space	3.161
C1qc	C1QC	Extracellular Space	3.269
Adam12	ADAM12	Plasma Membrane	3.167
C1qa	C1QA	Extracellular Space	3.503
Mmp14	MMP14	Extracellular Space	4.032
Thbs4	THBS4	Extracellular Space	4.578
Bmp1	BMP1	Extracellular Space	4.577
Npr1	NPR1	Plasma Membrane	5.607
Mtus2	MTUS2	Other	5.713
Syce1	SYCE1	Nucleus	7.905
4930447C04Rik	C14orf39	Extracellular Space	8.430

**FIGURE 5:**
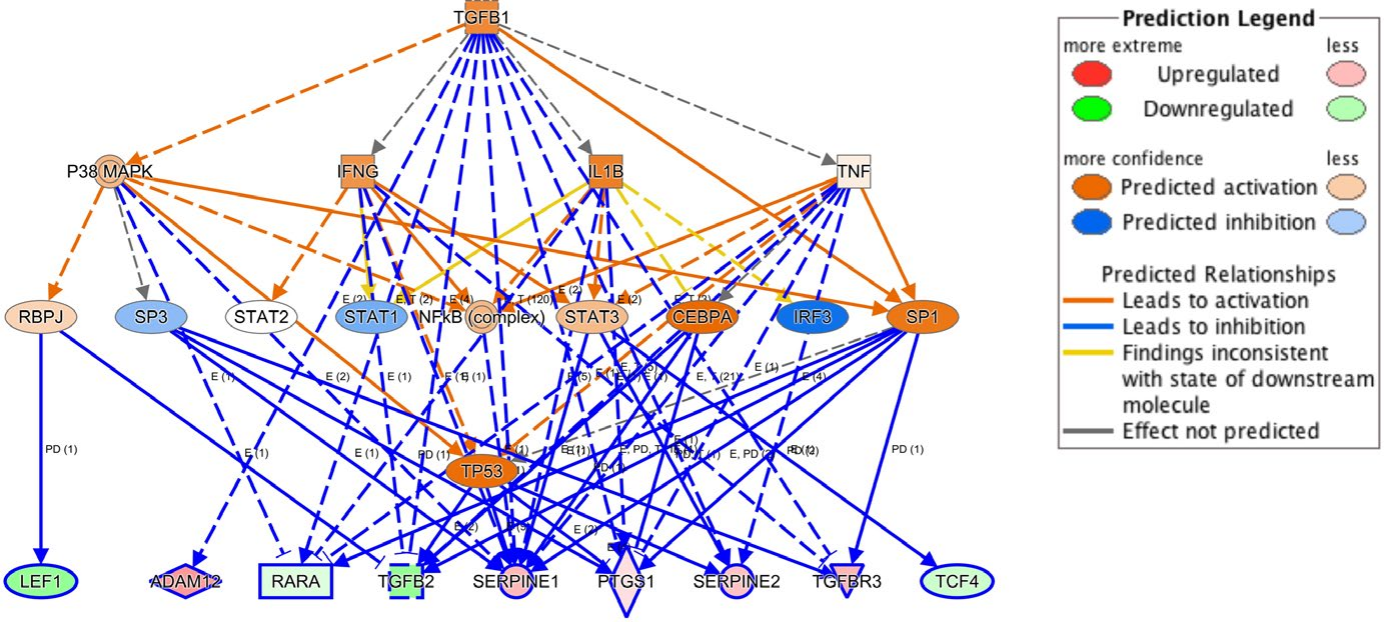
Upstream regulator analysis of differentially expressed genes. This analysis identified TGF‐β as a major regulator of differentially expressed genes (*P* = 8.52 × 10^−10^)

Ingenuity Pathway Analysis (IPA) allowed us to interrogate which upstream regulators contributed to gene expression changes. In this analysis, genes involved in TGF‐β signalling were significantly enriched (Figure 5). Other regulators identified by this analysis included all‐*trans* retinoic acid (ATRA) (*P* = 1.02 × 10^−8^) and serum response factor (SRF) (*P* = 5.34 × 10^−6^). Like TGF‐β targets, genes activated by ATRA were up‐regulated by *Tgif1* loss, consistent a role for Tgif1 as a corepressor of retinoic acid receptor (RAR)‐dependent transcription.

**FIGURE 6:**
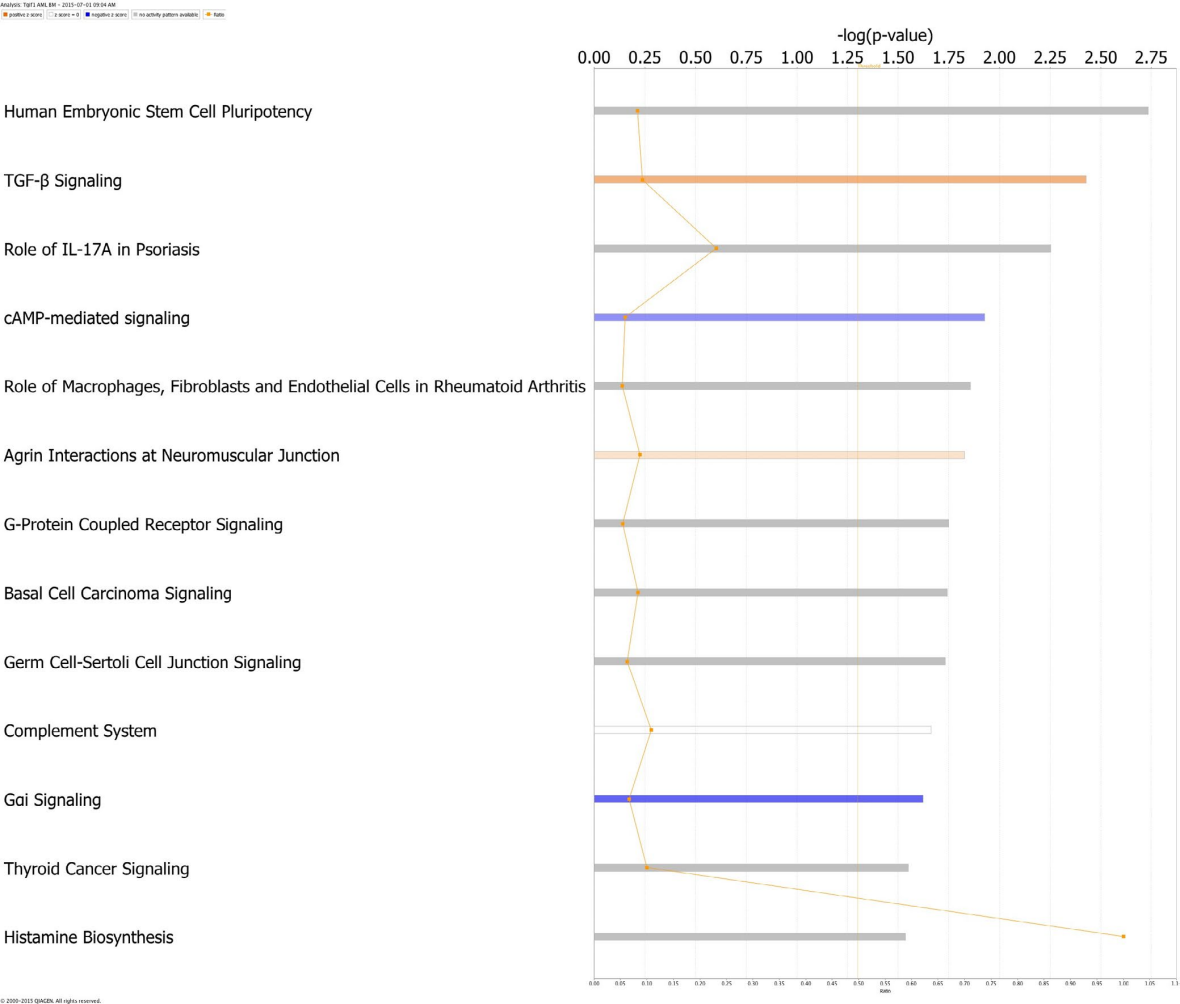
Canonical Pathway Analysis. Depicted are differentially utilized pathways in leukaemia cells from *Tgif1^−/−^* mice compared to *Tgif1^+/+^* mice

Genes important for embryonic stem cell pluripotency were highlighted in IPA canonical pathway analysis (*P* = 5.88 × 10^−4^) (Figure 6), while haematological system development and function (*P* = 1.05 × 10^−7^) were enriched in IPA physiological functions. Other functions impacted by *Tgif1* expression included cellular movement (*P* = 1.99 × 10^−9^), leukocyte function (*P* = 3.87 × 10^−8^), cell death and survival (*P* = 5.38 × 10^−8^), myeloid cell function (*P* = 3.24 × 10^−8^) and cancer (*P* = 2.63 × 10^−7^). Together, pathway analysis reveals altered regulation of TGF‐β signalling and RA signalling in *Tgif1^−/−^* leukaemia cells, with potentially important consequences for LICs function.

## 4 DISCUSSION

We have previously shown that inactivation of the *Tgif1* gene in mice, complete or partial, increases quiescence in bone marrow HSCs and enhances long‐term repopulating activity without effecting steady‐state haematopoiesis.^12^ We have also shown that *TGIF1* affects proliferation and differentiation of myeloid cell lines.^11^ Here, we sought to understand the role of *Tgif1* in mouse leukaemic haematopoiesis.

We find that loss or haploinsufficiency of *Tgif1* accelerates leukaemia development and shortens survival time in mouse models of MLL‐AF9‐induced AML and BCR‐ABL‐induced CML. AML in mice transplanted with oncogene‐transduced *Tgif1^−/−^* HSPCs shows an impaired response to conventional chemotherapy agents used to treat myeloid malignancy and correlates with earlier relapse and shorter survival compared to similarly transduced *Tgif1^+/+^* controls. In light of studies demonstrating that LICs frequency at diagnosis in AML correlates with increased minimal residual disease and poor survival,^20^ our finding that the *Tgif1* knockout enhances LIC functionality by increasing LIC frequency provides an explanation for how *Tgif1* expression affects AML recurrence in our model. Our results are well aligned with data showing that enforced expression of *TGIF1* decreases human leukaemia cell proliferation, induces terminal differentiation and increases survival in MLL‐AF9 rearranged myeloid leukaemia.^13^

In accordance with the transcriptional repression functions of TGIF1, mRNA expression profiling identified a number of genes which were up‐regulated in *Tgif1‐*null leukaemic cells, with almost half the genes differentially expressed between knockout and wild‐type leukaemia cells involved directly or indirectly in TGF‐β signalling (Figure 5). These results corroborate the enhanced TGF‐β signalling and/or target gene expression noted in epithelial ^6^ and myeloid leukaemia cells ^11^ with diminished *TGIF1* expression, and align with roles for TGF‐β in HSC self‐renewal,^21^ quiescence ^22^ and enhanced leukaemic stem cell function.^21^, ^35^

Loss of *Tgif1* function also relieves repression of retinoic acid receptor (RAR) target genes, consistent with its ability to act as a corepressor of RAR/RXR‐mediated transcription. Finally, our results do not exclude direct effects of *Tgif1* on gene expression or indirect effects through interaction with other TALE homeodomain proteins such as MEIS1^13^ As chemical inhibitors are available for TGF‐β and RAR signalling, it should be possible to dissect the unique contributions of these pathways to the altered *Tgif1* functions in leukaemia development.

Although rare loss‐of‐function variants in *TGIF1* are associated with holoprosencephaly,^9^, ^10^ these same *TGIF1* variants have not been observed in AML patients. Furthermore, individuals with holoprosencephaly and *Tgif1*‐null mice are not at increased risk for development of myeloid leukaemia, all of which argues against a function for this protein as a tumour suppressor. Interestingly Means‐Powell et al reported that *TGIF1* levels were an independent predictor of survival in AML, with lower levels associated with earlier relapse and poor survival^36^ (personal communication). Taken together, these data may suggest that *TGIF1* acts as a prototypical stem cell modifier gene, operating not in the initiation of leukaemia but in disease progression and persistence.

## CONFLICT OF INTEREST

The authors declare that they have no conflict of interest.

## AUTHOR CONTRIBUTION

**Ling Yan:** Data curation (lead); Investigation (lead); Methodology (lead); Project administration (lead); Writing‐original draft (lead); Writing‐review & editing (equal). **Utpal Dave:** Investigation (supporting); Methodology (supporting); Writing‐review & editing (supporting). **Michael Engel:** Data curation (supporting); Methodology (supporting); Writing‐review & editing (supporting). **Stephen Brandt:** Data curation (supporting); Project administration (supporting); Writing‐review & editing (supporting). **Rizwan Hamid:** Data curation (supporting); Funding acquisition (lead); Project administration (supporting); Supervision (lead); Writing‐review & editing (lead).

## References

[1] Hamid R , Patterson J , Brandt SJ . Genomic structure, alternative splicing and expression of TG‐interacting factor, in human myeloid leukemia blasts and cell lines. Biochim Biophys Acta. 2008;1779(5):347‐355. 18455519 10.1016/j.bbagrm.2008.04.003

[2] Wotton D , Knoepfler PS , Laherty CD , Eisenman RN , Massague J . The Smad transcriptional corepressor TGIF recruits mSin3. Cell Growth Differ. 2001;12(9):457‐463. 11571228

[3] Wotton D , Lo RS , Lee S , Massague J . A Smad transcriptional corepressor. Cell. 1999;97(1):29‐39. 10199400 10.1016/s0092-8674(00)80712-6

[4] Wotton D , Lo RS , Swaby LA , Massague J . Multiple modes of repression by the Smad transcriptional corepressor TGIF. J Biol Chem. 1999;274(52):37105‐37110. 10601270 10.1074/jbc.274.52.37105

[5] Bertolino E , Reimund B , Wildt‐Perinic D , Clerc RG . A novel homeobox protein which recognizes a TGT core and functionally interferes with a retinoid‐responsive motif. J Biol Chem. 1995;270(52):31178‐31188. 8537382 10.1074/jbc.270.52.31178

[6] Seo SR , Lallemand F , Ferrand N , et al. The novel E3 ubiquitin ligase Tiul1 associates with TGIF to target Smad2 for degradation. EMBO J. 2004;23(19):3780‐3792. 15359284 10.1038/sj.emboj.7600398 PMC522797

[7] Ming JE , Kaupas ME , Roessler E , et al. Mutations in PATCHED‐1, the receptor for SONIC HEDGEHOG, are associated with holoprosencephaly. Hum Genet. 2002;110(4):297‐301. 11941477 10.1007/s00439-002-0695-5

[8] Ming JE , Muenke M . Multiple hits during early embryonic development: digenic diseases and holoprosencephaly. Am J Hum Genet. 2002;71(5):1017‐1032. 12395298 10.1086/344412 PMC385082

[9] Wallis D , Muenke M . Mutations in holoprosencephaly. Hum Mutat. 2000;16(2):99‐108. 10923031 10.1002/1098-1004(200008)16:2<99::AID-HUMU2>3.0.CO;2-0

[10] Wallis DE , Muenke M . Molecular mechanisms of holoprosencephaly. Mol Genet Metab. 1999;68(2):126‐138. 10527664 10.1006/mgme.1999.2895

[11] Hamid R , Brandt SJ . Transforming growth‐interacting factor (TGIF) regulates proliferation and differentiation of human myeloid leukemia cells. Mol Oncol. 2009;3(5‐6):451‐463. 19699159 10.1016/j.molonc.2009.07.004 PMC5527533

[12] Yan L , Womack B , Wotton D , et al. Tgif1 regulates quiescence and self‐renewal of hematopoietic stem cells. Mol Cell Biol. 2013;33(24):4824‐4833. 24100014 10.1128/MCB.01076-13 PMC3889555

[13] Willer A , Jakobsen JS , Ohlsson E , et al. TGIF1 is a negative regulator of MLL‐rearranged acute myeloid leukemia. Leukemia. 2015;29(5):1018‐1031. 25349154 10.1038/leu.2014.307

[14] Hu Y , Smyth GK . ELDA: extreme limiting dilution analysis for comparing depleted and enriched populations in stem cell and other assays. J Immunol Methods. 2009;347(1‐2):70‐78. 19567251 10.1016/j.jim.2009.06.008

[15] Subramanian A , Tamayo P , Mootha VK , et al. Gene set enrichment analysis: a knowledge‐based approach for interpreting genome‐wide expression profiles. Proc Natl Acad Sci USA. 2005;102(43):15545‐15550. 16199517 10.1073/pnas.0506580102 PMC1239896

[16] Kirstetter P , Schuster MB , Bereshchenko O , et al. Modeling of C/EBPalpha mutant acute myeloid leukemia reveals a common expression signature of committed myeloid leukemia‐initiating cells. Cancer Cell. 2008;13(4):299‐310. 18394553 10.1016/j.ccr.2008.02.008

[17] Shlush LI , Zandi S , Mitchell A , et al. Identification of pre‐leukaemic haematopoietic stem cells in acute leukaemia. Nature. 2014;506(7488):328‐333. 24522528 10.1038/nature13038 PMC4991939

[18] Taussig DC , Vargaftig J , Miraki‐Moud F , et al. Leukemia‐initiating cells from some acute myeloid leukemia patients with mutated nucleophosmin reside in the CD34(‐) fraction. Blood. 2010;115(10):1976‐1984. 20053758 10.1182/blood-2009-02-206565 PMC2837317

[19] Zuber J , Radtke I , Pardee TS , et al. Mouse models of human AML accurately predict chemotherapy response. Genes Dev. 2009;23(7):877‐889. 19339691 10.1101/gad.1771409 PMC2666344

[20] van Rhenen A , Feller N , Kelder A , et al. High stem cell frequency in acute myeloid leukemia at diagnosis predicts high minimal residual disease and poor survival. Clin Cancer Res. 2005;11(18):6520‐6527. 16166428 10.1158/1078-0432.CCR-05-0468

[21] Pierelli L , Marone M , Bonanno G , et al. Transforming growth factor‐beta1 causes transcriptional activation of CD34 and preserves haematopoietic stem/progenitor cell activity. Br J Haematol. 2002;118(2):627‐637. 12139758 10.1046/j.1365-2141.2002.03604.x

[22] Yamazaki S , Iwama A , Takayanagi S , Eto K , Ema H , Nakauchi H . TGF‐beta as a candidate bone marrow niche signal to induce hematopoietic stem cell hibernation. Blood. 2009;113(6):1250‐1256. 18945958 10.1182/blood-2008-04-146480

[23] Falk LA , De Benedetti F , Lohrey N , et al. Induction of transforming growth factor‐beta 1 (TGF‐beta 1), receptor expression and TGF‐beta 1 protein production in retinoic acid‐treated HL‐60 cells: possible TGF‐beta 1‐mediated autocrine inhibition. Blood. 1991;77(6):1248‐1255. 1848114

[24] Imai Y , Kurokawa M , Izutsu K , et al. Mutations of the Smad4 gene in acute myelogeneous leukemia and their functional implications in leukemogenesis. Oncogene. 2001;20(1):88‐96. 11244507 10.1038/sj.onc.1204057

[25] Kim SJ , Letterio J . Transforming growth factor‐beta signaling in normal and malignant hematopoiesis. Leukemia. 2003;17(9):1731‐1737. 12970772 10.1038/sj.leu.2403069

[26] Krause DS , Fulzele K , Catic A , et al. Differential regulation of myeloid leukemias by the bone marrow microenvironment. Nat Med. 2013;19(11):1513‐1517. 24162813 10.1038/nm.3364 PMC3827980

[27] Kurokawa M , Mitani K , Imai Y , Ogawa S , Yazaki Y , Hirai H . The t(3;21) fusion product, AML1/Evi‐1, interacts with Smad3 and blocks transforming growth factor‐beta‐mediated growth inhibition of myeloid cells. Blood. 1998;92(11):4003‐4012. 9834202

[28] Kurokawa M , Mitani K , Irie K , et al. The oncoprotein Evi‐1 represses TGF‐beta signalling by inhibiting Smad3. Nature. 1998;394(6688):92‐96. 9665135 10.1038/27945

[29] Marone M , Scambia G , Bonanno G , et al. Transforming growth factor‐beta1 transcriptionally activates CD34 and prevents induced differentiation of TF‐1 cells in the absence of any cell‐cycle effects. Leukemia. 2002;16(1):94‐105. 11840268 10.1038/sj.leu.2402334

[30] Murohashi I , Endho K , Nishida S , et al. Differential effects of TGF‐beta 1 on normal and leukemic human hematopoietic cell proliferation. Exp Hematol. 1995;23(9):970‐977. 7543418

[31] Naka K , Hoshii T , Muraguchi T , et al. TGF‐beta‐FOXO signalling maintains leukaemia‐initiating cells in chronic myeloid leukaemia. Nature. 2010;463(7281):676‐680. 20130650 10.1038/nature08734

[32] Siegel PM , Massague J . Cytostatic and apoptotic actions of TGF‐beta in homeostasis and cancer. Nat Rev Cancer. 2003;3(11):807‐821. 14557817 10.1038/nrc1208

[33] Sitnicka E , Ruscetti FW , Priestley GV , Wolf NS , Bartelmez SH . Transforming growth factor beta 1 directly and reversibly inhibits the initial cell divisions of long‐term repopulating hematopoietic stem cells. Blood. 1996;88(1):82‐88. 8704205

[34] Soucek K , Pachernik J , Kubala L , Vondracek J , Hofmanova J , Kozubik A . Transforming growth factor‐beta1 inhibits all‐trans retinoic acid‐induced apoptosis. Leuk Res. 2006;30(5):607‐623. 16242776 10.1016/j.leukres.2005.09.007

[35] Tabe Y , Shi YX , Zeng Z , et al. TGF‐beta‐Neutralizing Antibody 1D11 Enhances Cytarabine‐Induced Apoptosis in AML Cells in the Bone Marrow Microenvironment. PLoS One. 2013;8(6):e62785. 23826077 10.1371/journal.pone.0062785 PMC3695026

[36] Means‐Powell JA , Kravtsov VSY , Levy SE , Greer JP , Koury MJ , Brandt S . Expression of homeobox gene TG‐interaction factor is an independent predictor of survival in acute myelogenous leukemia. 45th Am Soc Hematol Meet. 2003;764:p128a.

